# Ttc39c is a potential target for the treatment of lung cancer

**DOI:** 10.1186/s12890-022-02173-x

**Published:** 2022-10-27

**Authors:** Hao Rong, Jun Peng, Ke Ma, Jiang Zhu, Jin-Tao He

**Affiliations:** 1 Department of Thoracic Surgery, Sichuan Cancer Hospital & Institute, No. 55, 4th section, South Renmin Road, 610054 Chengdu, Sichuan China; 2 Sichuan Cancer Center, School of Medicine, No. 55, 4th section, South Renmin Road, 610054 Chengdu, China; 3 University of Electronic Science and Technology of China, No. 55, 4th section, South Renmin Road, 610054 Chengdu, China

**Keywords:** Ttc39c, Lung adenocarcinoma, Bioinformatics, TPR

## Abstract

**Background:**

The novel TTC gene, tetratricopeptide repeat domain 39 C (Ttc39c), mainly mediates the interaction between proteins. It is involved in the progression of various tumors. In this study, we determined the effect of Ttc39c on lung adenocarcinoma and found that it might be used as a potential intervention target.

**Methods:**

We performed a difference analysis of Ttc39c samples from the TCGA database. Transwell experiments were conducted to determine the ability of cell metastasis. Celigo and MTT assays were performed to determine the effect of Ttc39c gene subtraction on cell proliferation. FACS was performed to determine the effect of Ttc39c gene subtraction on apoptosis. Clone-formation experiments were conducted to determine the effect of Ttc39c gene subtraction on cloning ability. Transcriptomics, proteomics, and metabolomics were used to elucidate the enrichment pathway of the Ttc39c gene in the progression of lung adenocarcinoma.

**Results:**

The expression of Ttc39c increased significantly in lung adenocarcinoma. The proliferation, metastasis, and cloning ability of human lung cancer cells were inhibited, while the apoptosis of cells increased significantly after the depletion of Ttc39c. Our results based on the transcriptomics, proteomics, and metabolomics analyses indicated that Ttc39c might be involved in the progression of lung adenocarcinoma (LUAD) mainly through the metabolic pathway and the p53 pathway.

**Conclusion:**

To summarize, Ttc39c strongly regulates the proliferation and metastasis of lung adenocarcinoma cells. The main pathways involved in Ttc39c in lung adenocarcinoma include the energy metabolism and p53 pathways.

**Supplementary information:**

The online version contains supplementary material available at 10.1186/s12890-022-02173-x.

## Introduction

Lung cancer has one of the highest rates cancers in the world [1], although its the cure rate of it is improving. Most lung cancer patients are treated in the late stage because they are asymptomatic in the early stages [2]. Lung adenocarcinoma (LUAD) is the most common subtype of lung cancer, and its incidence rate is increasing every year [3, 4]. This might be due to an increase in the number of smokers [5]. LUAD is more common among women (especially Asian women) than among men [6, 7]. It is more likely to occur than other types of lung cancer in young people [8]. Besides smoking, external environmental factors and genetic changes, including abnormal activation of signal pathways caused by gene amplification, mutation, or translocation, can promote the progression of lung adenocarcinoma [9–11]. Molecular targeted therapy and immunotherapy are mainly administered for treating lung cancer. The interaction between PD-L1 and programmed death receptor-1 (PD-1) on lung cancer cells inhibits signal transduction [12]. This interaction can effectively inhibit the response of T cells to tumors. Monoclonal antibodies are also administered to patients with LUAD for immunomodulatory therapy [13, 14]. Although they can significantly improve the survival of the patients, the cure for lung cancer is still challenging. Therefore, new therapeutic targets might facilitate the treatment of lung adenocarcinoma.

The TTC proteins participate extensively in the formation of protein complexes and important biological processes, such as intracellular transport, vesicle fusion, protein folding, cell cycle, and transcriptional regulation. The novel TTC gene, tetratricopeptide repeat domain 39 C (Ttc39c), is a protein-coding gene located on the long arm of chromosome 18 [15, 16]. The expression of the Ttc39c gene is significantly upregulated in STK11 mutant lung cancer tissue samples [17]. The protein encoded by the Ttc39c gene contains several putative tetrapeptide repeat (TPR) domains, which form a structural motif that promotes protein-protein interactions [18]. The TPR domain affects cell cycle regulation and signal domain [19, 20]. Lung cancer can activate the host immune response and trigger antibodies against tumor antigens (AAbs). The mRNA of tetratricopeptide repeat domain 14 (Ttc14) is a target of AABS and is highly expressed in LUAD [21]. We speculated that Ttc39c might mediate the interaction between proteins in patients with LUAD through the special protein space structure of the TPR domain.

In this study, we analyzed the changes in the expression of Ttc39c in patients with lung adenocarcinoma using information from the TCGA database. Moreover, we found that the survival rate of lung adenocarcinoma patients with high expression of Ttc39c was significantly lower. The proliferation rate of A549 and NCI-H1299 cells was significantly inhibited after Ttc39c was knocked out, and the metastatic ability of the two cells was also significantly inhibited. Additionally, the apoptosis of the A549 and NCI-H1299 cells increased, and their clonogenic ability decreased. Furthermore, transcriptomics, proteomics, and metabolomics analyses showed that the Ttc39c gene might affect the progression of lung adenocarcinoma by mediating cell energy transport and signal transduction pathways.

## Methods

### Cell transfection

The A549 cells were purchased in CAS, China, and the NCI-H1299 cells were purchased in ATCC, USA. The A549 cells were cultured in the F12K medium (Invitrogen, 21,127–022) supplemented with 10% fetal bovine serum (Ausbian VS500T). The NCI-H1299 cells were cultured in the 1640 medium (Corning, 10–040-CVB), supplemented with 10% fetal bovine serum. The A549 and NCI-H1299 cells grown in the logarithmic phase were inoculated in six-well plates. The A549 cells were transfected with 10 µL shTtc39c/shCtrl (Genechem, China), and the NCI-H1299 cells were transfected with 5 µL shTtc39c/shCtrl, following the manufacturer’s instructions. The medium was replaced by the complete medium after 8–16 h. The expression of the reporter genes (such as GFP) was determined using a fluorescence microscope about 72 h after infection, and the fluorescence rate indicated a positive infection rate. All methods were performed following relevant guidelines.

### Animal model of LUAD

Female BALB/c nude mice (20–22 g, four weeks old) were purchased from Beijing Wei Tong Li Hua Laboratory Animal Technology Co., Ltd. All animal experiments were approved by the Animal Experimental Ethics Committee (Number: GSGC0291706). The animal experiments were conducted at the Animal Experiment Center in (No.332 Edison Road). The mice were kept in a controlled environment at a constant temperature (25 ± 1 °C) and humidity (50 ± 5%) environment with a 12 h/12 h light/dark cycle and ad libitum access to food and water. The mice were divided into the NC and KD groups. The two groups of mice were infected with shCtrl / shTtc39c lentivirus respectively. The sequence for shTtc39c was: CCGGCGTCTATTGAAGTGTTGTACTCTCGAGAGTACAACACTTCAATAGACGTTTTT. The A549 tumor cells were prepared (2e + 7 / ml) and injected subcutaneously into the animals (200 µL/mouse). The tumor-forming condition was observed based on the tumor-forming ability of the cells. The tumor size and the weight of the animals were measured after 5–20 days. After 28 days of subcutaneous injection, the mice were euthanized by excessive injection of 2% pentobarbital sodium. Cervical spondylolisthesis was performed to confirm that they were dead. The tumor and lung tissue samples were isolated for the experiments. At least six independent experiments were performed. All methods were conducted following relevant guidelines.

### Real-time qPCR

Total cellular RNA was extracted using the Trizol kit (Pufei, Shanghai). The Nanodrop 2000/2000c spectrophotometer was used to analyze the samples and determine the concentration and quality of extracted RNA (Thermo Scientific, USA). Reverse transcription using the Promega m-mlv kit (Axygen, USA) was performed to obtain cDNA. Then, the reaction system was configured in proportion, and real-time PCR was performed. The primers used were synthesized by Guangzhou Ruibo Biotechnology Co., Ltd. (http://www.ribobio.com/), and their sequences are listed in Table 1. All methods were conducted following relevant guidelines.

**Table 1:** The primer sequences used to perform real-time PCR

Genes	Forward primer (5′–3′)	Reverse primer (5′–3′)
Gapdh	TGACTTCAACAGCGACACCCA	CACCCTGTTGCTGTAGCCAAA
Ttc39c	ATGCCATGATGACATTTGAGGAA	GGGGCGGATTTTCGGACAT

### Western blotting

Lung adenocarcinoma tissues were lysed with the RIPA lysis buffer (Biyuntian, China). The protein concentration was measured using the BCA protein assay kit (Boster, China), and the extracted protein samples were separated by 10% or 12% SDS–polyacrylamide gel and transferred onto polyvinyl difluoride membranes (Millipore, USA). After blocking, the membranes were incubated overnight at 4 °C with the primary antibodies of β-actin (1:5000) and Ttc39c (1:3000) for 24 h, followed by incubation with the secondary antibody (1:10000) for 1 h at room temperature. The membranes were visualized using an ECL-chemiluminescent kit (ECL-plus, CST) and exposed to a medical X-ray machine (Carestream, 038401501). The intensities of the bands were quantified using the Image J software (NIH, Bethesda, MD, USA). All methods were conducted following relevant guidelines.

### Fluorescence activating cell sorter

The cells were collected and centrifuged. Then, the precipitate was washed and resuspended in a buffer. Next, 10 µL of Annexin V-APC (Invitrogen, 88–8007–74) stain was added and incubated in the dark at room temperature for 10–15 min. Based on the cell quantity, 400–800 µL of 1 × Binding buffer was added and detected using the machine. All methods were conducted following relevant guidelines.

### Cell clone-formation assay

The infected cells were inoculated in a six-well plate and placed in an incubator for further culture until 14 days or till the number of cells in most single clones was greater than 50. The cell clones were photographed using a fluorescence microscope before the end of the experiment. Next, 1,000 µL of clean and impurity-free crystal violet (Shanghai, China) dye was added to each well after fixing the cells. The cells were stained for 10–20 min and washed using ddH2O several times. They were dried, photographed with a digital camera, and counted. All methods were conducted following relevant guidelines.

### Transwell

The transfer kit (Corning, USA) was taken out, the required number of chambers was placed in a new 24-well plate, and 100 µL of the serum-free medium was added to the upper chamber and placed in the incubator for 1 h. The medium in the upper chamber was removed carefully, and 100 µL of cell suspension was added, followed by the addition of 600 µL of 30% FBS medium in the lower chamber. The cell plates were incubated in an incubator at 37 °C for some time. The chamber was taken out and fixed. Then, 1–2 drops of the staining solution were added to the lower surface of the membrane; the cells were stained for 1–3 min and transferred. Then, the chamber was soaked and rinsed several times and air dried. Finally, photographs were taken using a microscope. All methods were conducted following relevant guidelines.

### Celigo scratch test

The cells were incubated in 96-well plates. To perform the scratch test, the scratch instrument was aligned with the central part of the upper end of the 96-well plate and gently pushed upward to form a scratch. The plates were gently rinsed with PBS 2–3 times, the medium containing 1% FBS serum was added, and the plates were swept for 0 h. A suitable time was selected to sweep the plates with Celigo based on the degree of healing, and samples were collected at three time points. Finally, the migration area was analyzed by Celigo. All methods were conducted following relevant guidelines.

### Transcriptome analysis

We selected 6 NC animal samples and 6 KD animal samples for conducting the transcriptome analysis. The integrity of RNA was detected using the Agilent 2100 Bioanalyzer after RNA extraction. Approximately 1 µg of RNA per sample was used as the input material for preparing the RNA samples. Briefly, mRNA was purified from total RNA using poly-T oligo-attached magnetic beads. Fragmentation was conducted using divalent cations at elevated temperatures using the First Strand Synthesis Reaction Buffer (5 X). First-strand cDNA was synthesized using random hexamer primers and the M-MuLV Reverse Transcriptase (RNase H). Second-strand cDNA was synthesized using DNA Polymerase I and RNase H. The remaining overhangs were converted into blunt ends via exonuclease/polymerase activities. After adenylation of the 3’ ends of the DNA fragments, adaptors with hairpin loops were ligated for hybridization. To select cDNA fragments that were 370 ~ 420 bp long, the library fragments were purified using the AMPure XP system (Beckman Coulter, Beverly, USA). Then, PCR was performed using the Phusion High-Fidelity DNA polymerase, Universal PCR primers, and the Index (X) Primer. Finally, the PCR products were purified (AMPure XP system), and the quality of the library was assessed using the Agilent Bioanalyzer 2100 system. The clustering of the index-coded samples was performed on a cBot Cluster Generation System using the TruSeq PE Cluster Kit v3-cBot-HS (Illumina), following the manufacturer’s instructions. After the clusters were generated, the library preparations were sequenced on an Illumina NovaSeq platform, and 150 bp paired-end reads were generated. All methods were conducted following relevant guidelines.

### Proteomics analysis

SDT buffer was added to the sample, and transferred to 2 ml tubes with amount quartz sand. The lysate was homogenized by MP Fastprep-24 Automated Homogenizer (6.0 M/S, 30s, twice). The homogenate was sonicated and then boiled for 15 min. After centrifuged at 14,000 g for 40 min, the supernatant was filtered with 0.22 μm filters. The filtrate was quantified with the BCA Protein Assay Kit (P0012, Beyotime). The sample was stored at -20 °C. 20 µg of proteins for each sample were mixed with 6X loading buffer respectively and boiled for 5 min. The proteins were separated on 12.5% SDS-PAGE gel. Protein bands were visualized by Coomassie Blue R-250 staining. 200 µg of proteins for each sample were incorporated into 30 µl SDT buffer (4% SDS, 100 mM DTT, 150 mM Tris-HCl pH 8.0). The detergent, DTT and other low-molecular-weight components were removed using UA buffer (8 M Urea, 150 mM Tris-HCl pH 8.5) by repeated ultrafiltration (Sartorius, 30 kD). Then 100 µl iodoacetamide (100 mM IAA in UA buffer) was added to block reduced cysteine residues and the samples were incubated for 30 min in darkness. The filters were washed with 100 µl UA buffer three times and then 100 µl 0.1 M TEAB buffer twice. Finally, the protein suspensions were digested with 4 µg trypsin (Promega) in 40 µl 0.1 M TEAB buffer overnight at 37 °C, and the resulting peptides were collected as a filtrate. The peptide content was estimated by UV light spectral density at 280 nm using an extinctions coefficient of 1.1 of 0.1% (g/l) solution that was calculated on the basis of the frequency of tryptophan and tyrosine in vertebrate proteins. 100 µg peptide mixture of each sample was labeled using TMT reagent according to the manufacturer’s instructions (Thermo Fisher Scientific). Then, the Agilent 1260 infinity II HPLC system was used for grading. Finally, the sample was analyzed by mass spectrometry and data processing.

### Gene Ontology (GO) annotation

At first, all protein and gene sequences were aligned to database downloaded from NCBI, only the sequences in top 10 and E-value < = 1e-3 were kept. Secondly, the GO term (database version: go_201504.obo) of the sequence with top Bit-Score by Blast2GO was selected. Then, the annotation from GO terms to proteins was completed by Blast2GO Command Line. After the elementary annotation, InterProScan were used to search EBI database by motif and then add the functional information of motif to proteins to improve annotation. Fisher’s Exact Test were used to enrich GO terms by comparing the number of differentially expressed proteins and differentially genes correlated to GO terms.

### KEGG pathway annotation

In KEGG database, KO (KEGG Orthology) is a classification system for genes and their products. Direct homologs with similar functions in the same pathway and their products are grouped together and assigned the same KO (or K) tag. When KEGG pathway Annotation of target protein set was carried out, KOALA (KEGG Orthology And Links Annotation) software was used to classify target protein sequences by KO by comparing KEGG GENES database. The pathway information involved in the target protein sequence was automatically obtained according to KO classification.

### Enrichment analysis of GO annotation and KEGG annotation

In the enrichment analysis of GO annotation or KEGG pathway annotation for the target protein set, Fisher’s Exact Test was used to compare the distribution of each GO classification or KEGG pathway in the target protein set and to evaluate the significance level of protein enrichment degree of a GO term or KEGG pathway.

### Metabonomic analysis

To a suitable amount of the sample, precooled methanol/acetonitrile/aqueous solution was added (2:2:1, V/V) after thawing the samples at 4 °C. The samples underwent low-temperature ultrasound treatment for 30 min after vortex-mixing. Then, they were left undisturbed at − 20 °C for 10 min, after which they were centrifuged (14,000 g) at 4 °C for 20 min. The supernatant was collected and dried in a vacuum chamber. Next, 100 µL of acetonitrile aqueous solution (acetonitrile: water = 1:1, V/V) was added to perform mass spectrometry. The samples were vortexed and centrifuged for 15 min. Finally, the supernatant was analyzed by chromatography-mass spectrometry. The samples were separated using a 1290 infinity LC ultra-high-performance liquid chromatography system (UHPLC) (Agilent, USA) equipped with a HILIC column. An AB triple TOF 6600 mass spectrometer (AB SCIEX, USA.) was used to collect the primary and secondary spectra of the samples. Finally, data analysis and quality evaluation were conducted. All methods were conducted following relevant guidelines.

### Statistical analysis

All experimental data are presented as the mean ± standard deviation of independent experiments repeated at least thrice. Statistical analysis was performed using SPSS version 22.0. One-way analysis of variance (ANOVA) and unpaired two-tailed t-tests were performed to evaluate the differences between groups. Significant enrichment analysis was statistically analyzed by performing Fisher’s exact tests. Fuzzy c-means (FCM) algorithm was used to analyze the metabolites. The OPLS-DA model was used to screen differential metabolites. All differences were considered to be statistically significant at *P* < 0.05.

## Results

Ttc39c **was increased in lung adenocarcinoma**.

**Fig. 1:**
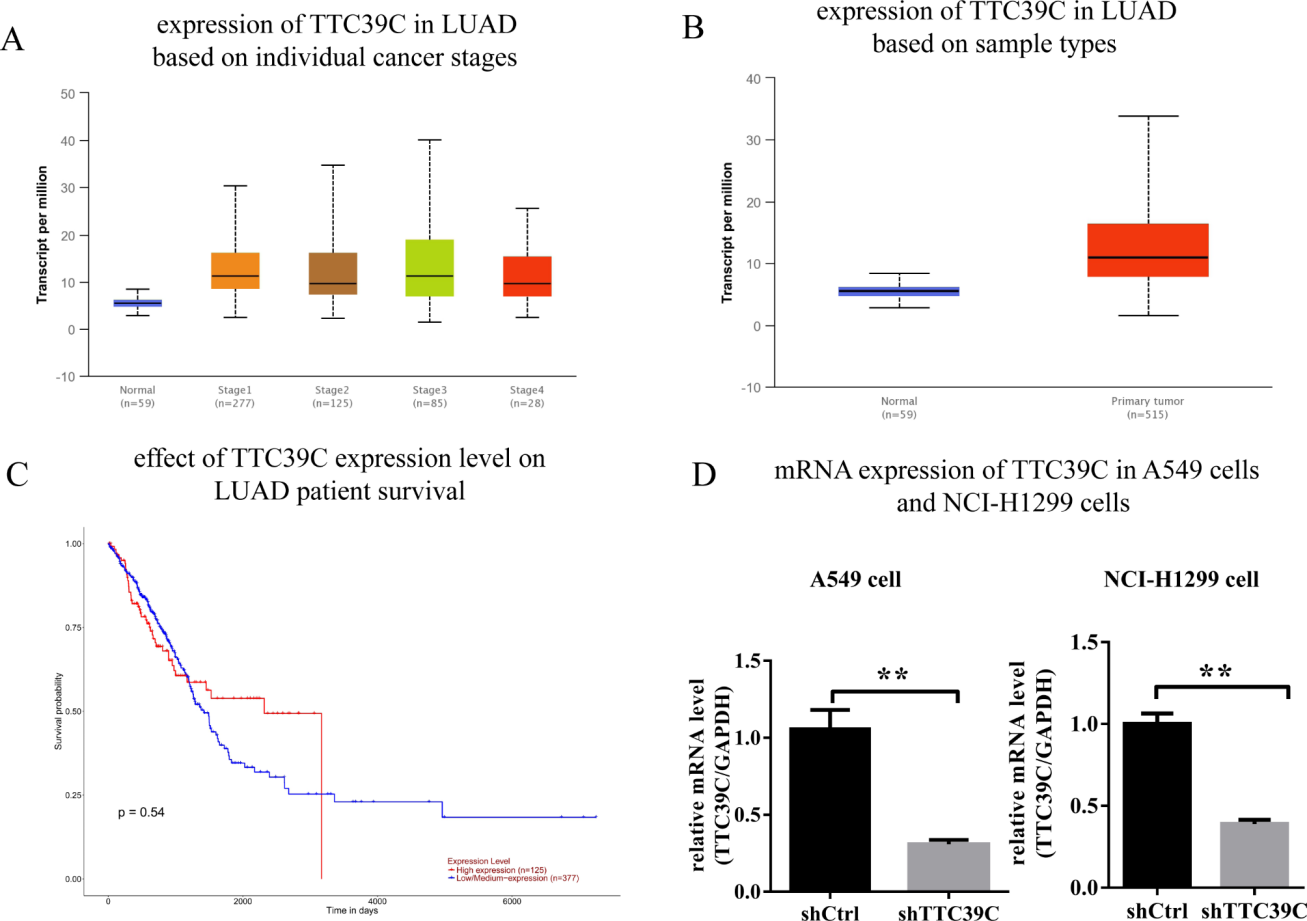
The expression of Ttc39c in patients with lung adenocarcinoma was analyzed by TCGA database. (A) The expression of Ttc39c in LUAD based on individual cancer stages. (B) The expression of Ttc39c in LUAD based on sample types. (C) The effect of Ttc39c expression level on LUAD patient survival. (D) The mRNA expression of Ttc39c in A549 cells and NCI-H1299 cells (n > 3). Bar represents the mean ± SEM. Signifcance *P < 0.05, **P < 0.01 vs. shTtc39c group

Data on 59 normal tissues, 277 stage 1 LUAD, 125 stage 2 LUAD, 85 stage 3 LUAD, and 28 stage 4 LUAD were downloaded from the TCGA database. The expression of Ttc39c in LUAD tissues was significantly higher than that in the normal tissues (Fig. 1 A). The expression of Ttc39c in the LUAD samples was significantly higher than that in the normal samples (Fig. 1B). We also compared the survival rates of the LUAD patients with higher Ttc39c expression to the survival rates of those patients with low or moderate expression of Ttc39c. Data from the TCGA database on 125 patients with low or moderate expression of Ttc39c and 377 patients with high expression of LUAD were analyzed. The results showed that the LUAD patients with high expression of Ttc39c had lower survival rates, and the 10-year survival probability of the patients with high expression of LUAD was almost zero (Fig. 1 C). These results suggested that Ttc39c might play an important role in the development and treatment of LUAD.

### Downregulation of Ttc39c inhibits the proliferation and metastasis of A549 and NCI-H1299 cells

**Fig. 2:**
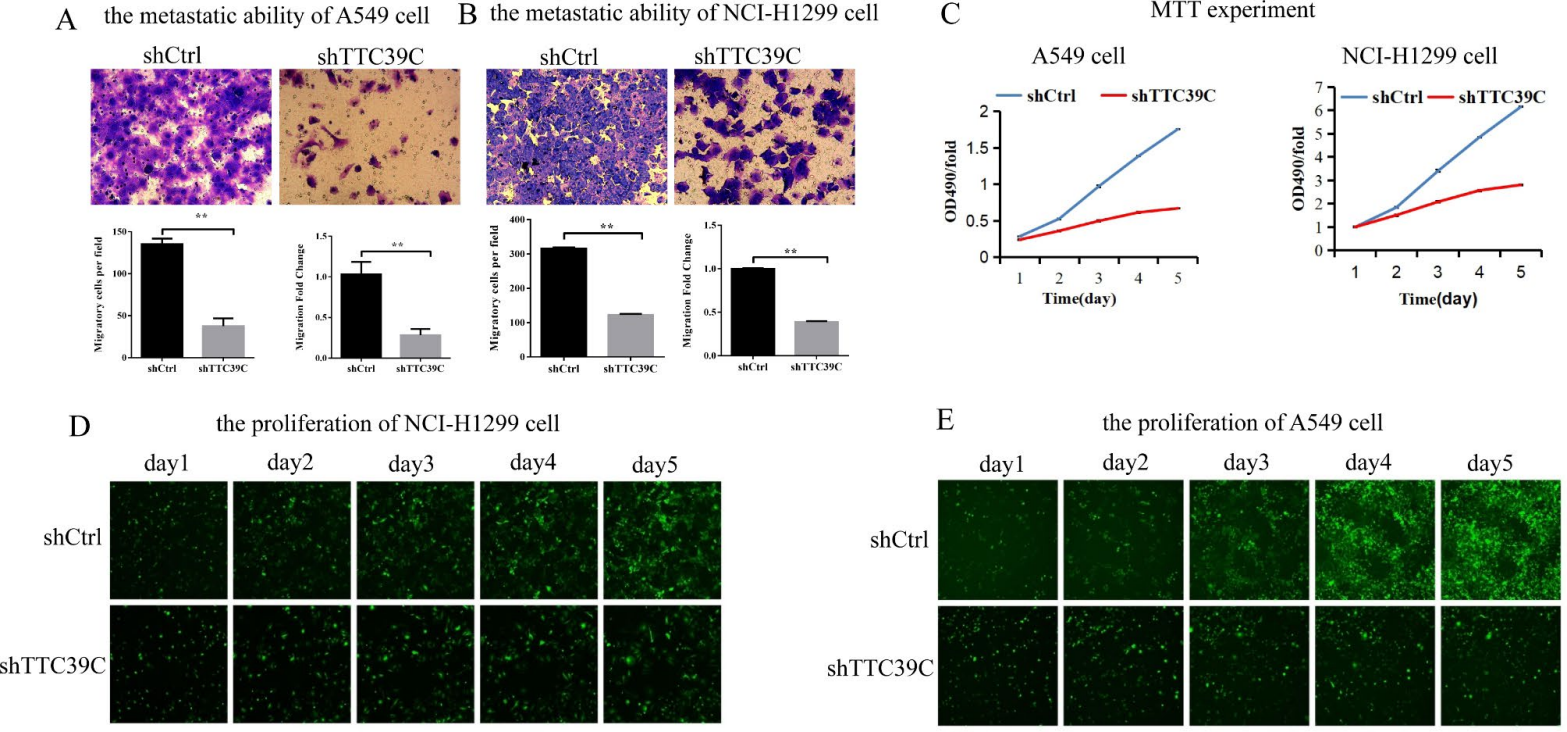
Down regulation of Ttc39c inhibits the proliferation and metastasis in A549 and NCI-H1299 cells. (A) The metastatic ability of A549 cell. (B) The metastatic ability of NCI-H1299 cell (n = 3). (C) MTT experiment of A549 cells and NCI-H1299 cells. (D) the proliferation of NCI-H1299 cell (n = 3, scale bars = 100 μm). (E) the proliferation of A549 cell (n = 3, scale bars = 100 μm). Bar represents the mean ± SEM. Signifcance *P < 0.05, **P < 0.01 vs. shTtc39c group

To determine the effect of Ttc39c on the proliferation and migration of lung cancer cells, we selected the human lung cancer cell lines A549 and NCI-H1299 for the experiments. Infection with the shRNA lentivirus was performed to knock down the Ttc39c gene. After three days of infection, the mRNA level of the Ttc39c gene was reduced in the A549 and NCI-H1299 cells in the experimental group (Fig. 1D). Then, the effect of Ttc39c gene subtraction on cell metastasis was determined by the Transwell method without ECM. The metastatic ability of the A549 and NCI-H1299 cells in the experimental group was significantly inhibited compared to that in the control group (Fig. 2 A and 2B). Additionally, the MTT assay was performed to examine the proliferation of the two types of cells for five days. The results showed that the proliferation of the A549 and NCI-H1299 cells decreased significantly after the Ttc39c gene was knocked down (Fig. 2 C). The results of Celigo imaging were consistent with those of the MTT assay (Fig. 2D and E). This suggested that the Ttc39c gene was strongly associated with the proliferation and metastasis of the A549 and NCI-H1299 cells.

**Effects of Ttc39c knockdown on the clonogenic ability and apoptosis of the A549 and NCI-H1299 cells**.

**Fig. 3:**
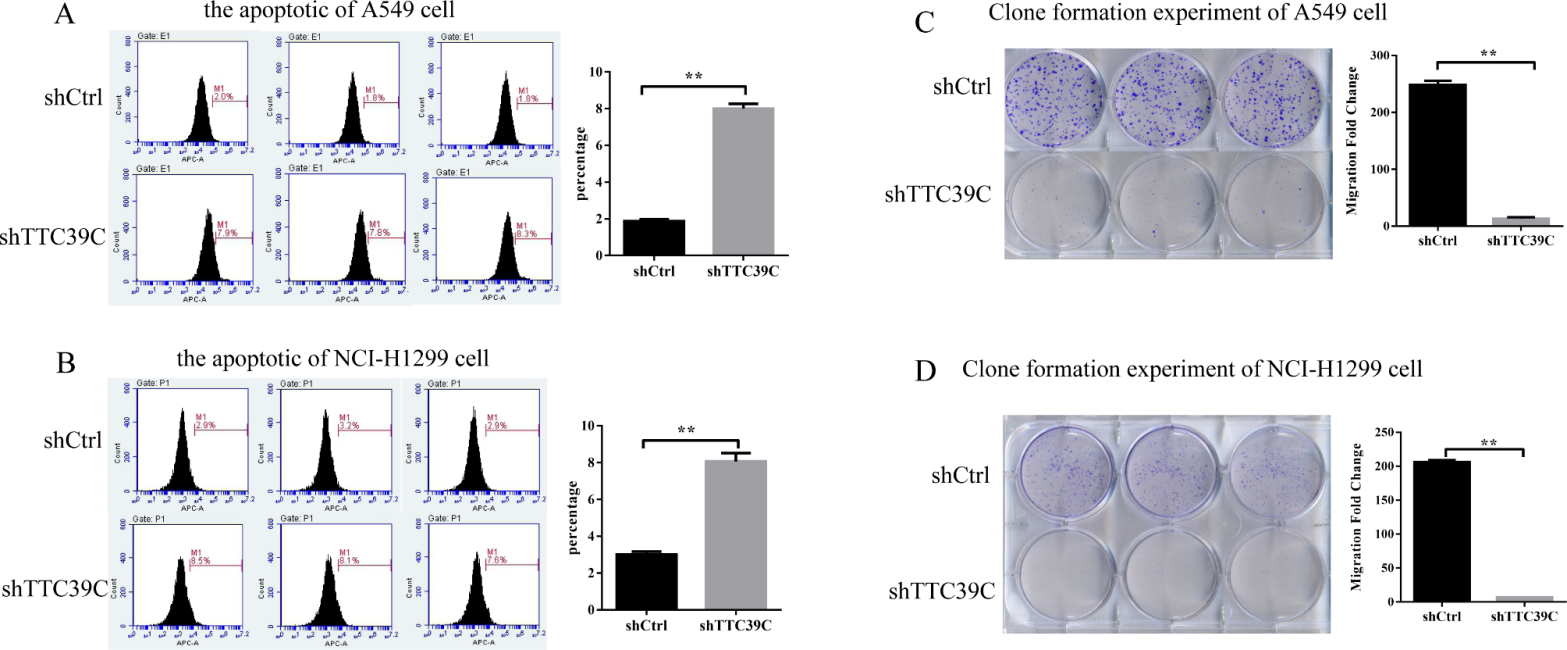
The effects of Ttc39c knockdown on cell clonogenic ability and apoptosis in A549 and NCI-H1299 cells. (A) the apoptotic of A549 cell (n = 3). (B) the apoptotic of NCI-H1299 cell (n = 3). (C) Clone formation experiment of A549 cell (n = 3). (D) Clone formation experiment of NCI-H1299 cell (n = 3). Bar represents the mean ± SEM. Signifcance *P < 0.05, **P < 0.01 vs. shTtc39c group

The apoptosis and the cloning ability of cancer cells are closely related to the prognosis of cancer. Therefore, we examined the relationship of Ttc39c with cell apoptosis and the cloning ability. Apoptosis was detected by performing single staining using Annexin V-APC. The results showed that the number of apoptotic A549 and NCI-H1299 cells in the experimental group increased significantly after five days of infection with the shRNA lentivirus (Fig. 3 A and 3B). The clone formation experiment showed that the number of A549 and NCI-H1299 cell colonies decreased after Ttc39c knockdown (Fig. 3 C and 3D). We found that the Ttc39c gene was significantly related to the apoptosis and cell clonogenic ability of the A549 and NCI-H1299 cells.

**Transcriptome and proteomic analysis of lung adenoma samples from** Ttc39c**- knockout and normal nude mice**.

**Fig. 4:**
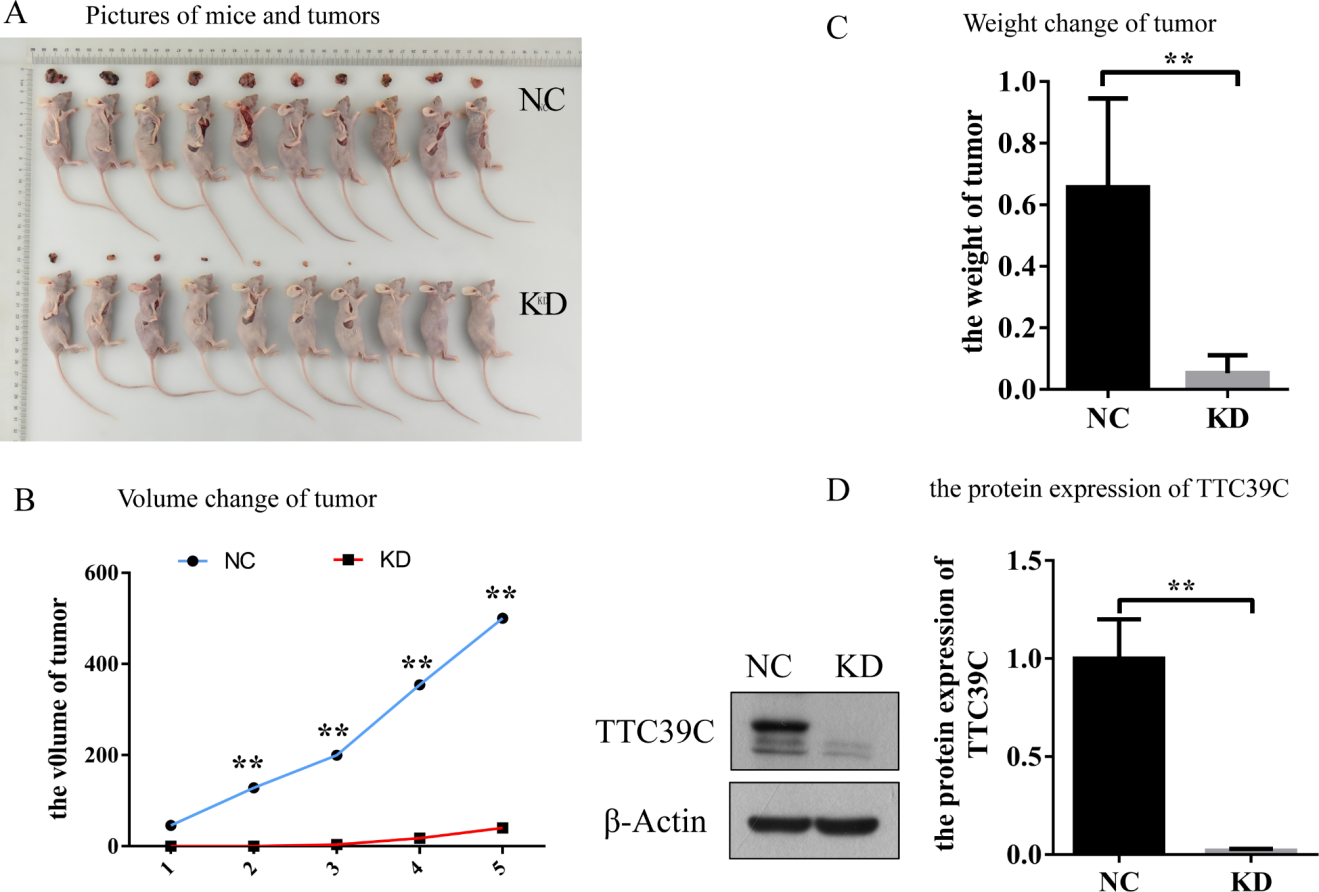
The effect of Ttc39c gene knockout on LUAD. (A) Representative image of mice and tumors in NC and KD group (n = 10). (B) The volume change of tumor in NC and KD group (n = 6). (C) The weight change of tumor in NC and KD group (n = 6). (D) The protein expression of Ttc39c in NC and KD group (n = 6). Bar represents the mean ± SEM. Signifcance *P < 0.05, **P < 0.01 vs. shTtc39c group

The Ttc39c gene strongly affects the proliferation and migration of LUAD. Thus, we determined the involvement of TTC in the progression of LUAD at the gene and protein levels. Female BALB/c nude mice (n = 10) and Ttc39c-knockdown transgenic BALB/c nude mice (n = 10) were used for the experiment. The A549 human lung cancer cells were injected into the two groups of mice via the right skin axillary. Tumor formation in the mice was observed after 39 days. The tumors of the mice in the NC group were significantly larger than those of the mice in the KD group (Fig. 4 A and 4B), and the tumor body increased with the test cycle. The average weight of the tumor in the NC group was significantly higher than that of the tumor in the KD group (Fig. 4 C). RNA sequencing was performed for database construction, sequencing, and biological information analysis. The heatmap analysis of the gene expression profile showed that the profile had a relatively good consistency and tendency in the mice of the NC and KD groups, respectively (Additional Fig. 1 A). The PCA plots showed distinct segregation between the mice of the NC and KD groups, indicating that the gene expression profile could be used to differentiate the mice of the NC group from those of the KD group (Additional Fig. 1B). Consistent with these findings, the protein samples in the groups also showed good repeatability (Additional Fig. 1 C and 1D).

### Analysis of the biological information related to proteome and transcriptome

**Fig. 5:**
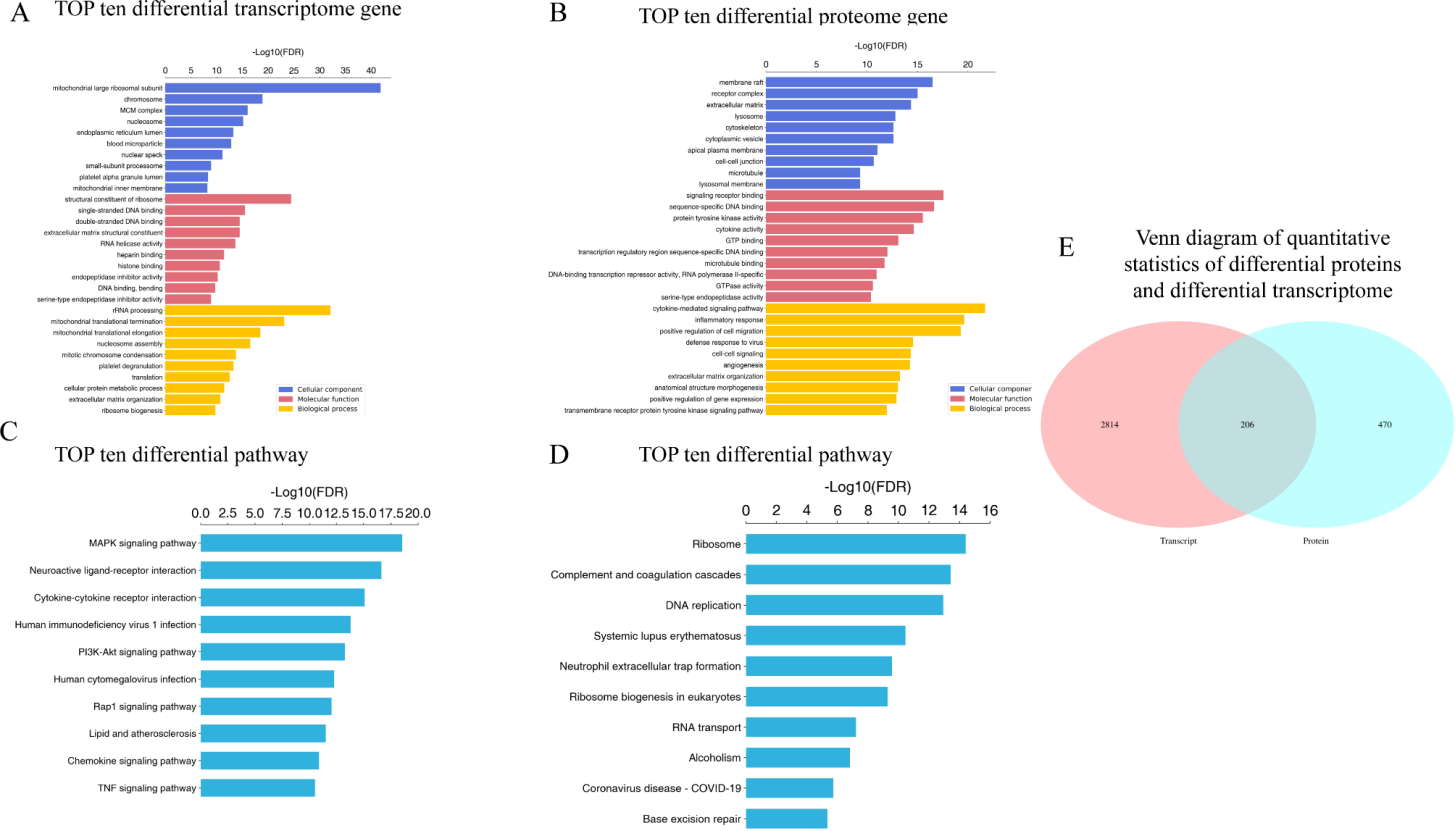
Analysis of biological information related to proteome and transcriptome. (A) The GO enrichment analyses the top 10 different genes proteins between the two groups. (B) The GO enrichment analyses the top 10 different proteins between the two groups. (C) KEGG enrichment analyses the top 10 differentially pathway with the most significant enrichment degree among genes participate in LUAD. (D) KEGG enrichment analyses the top 10 differentially pathway with the most significant enrichment degree among protein participate in LUAD. The abscissa is the enrichment significance level FDR (negative logarithmic transformation based on 10). The higher the ranking, the more statistically significant the influence or change of this pathway. (E) Venn plot shows the corresponding coincidence distribution between proteome and transcriptome

**Fig. 6:**
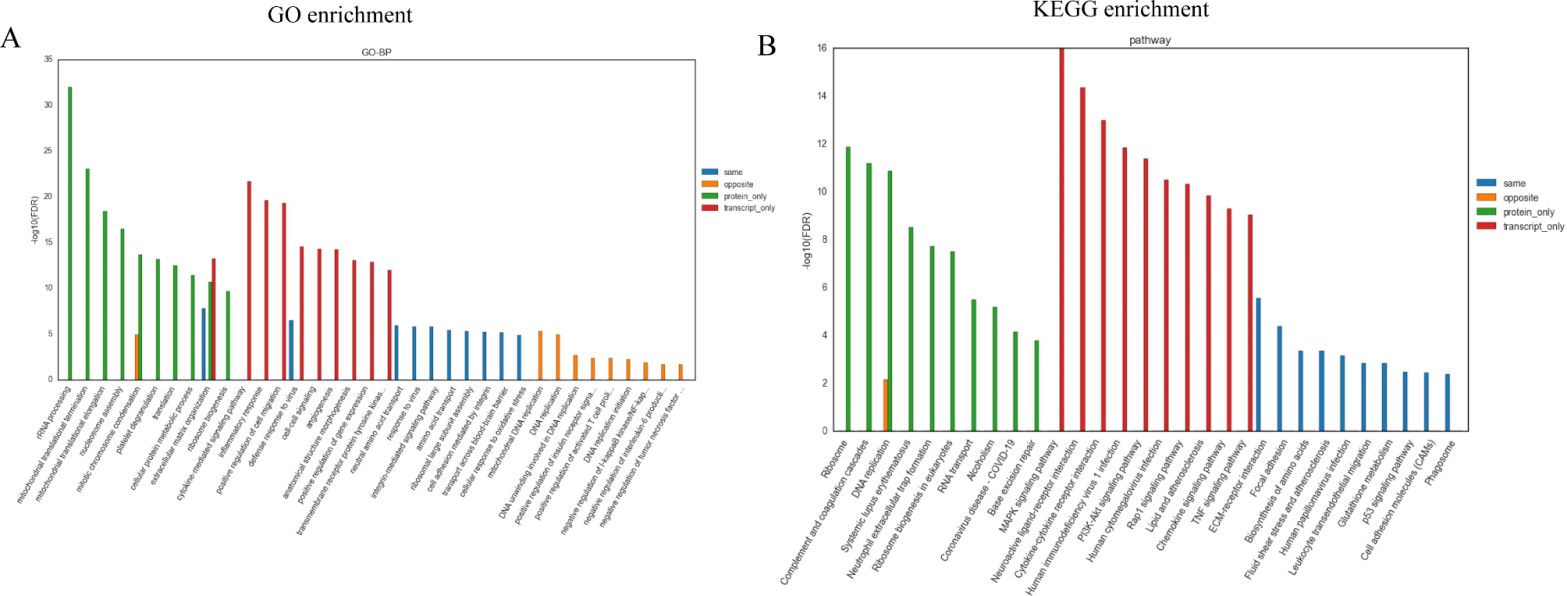
Combined analysis of transcriptome and proteome. GO (A) and KEGG (B) enrichment analyses the top 10 biological process entries in the four types of association analysis results of proteome and transcriptome. The abscissa is the biological process of significant enrichment, and the ordinate is the significance level FDR of enrichment. Same indicates the enrichment analysis results of genes with the same expression trend in protein group and transcriptome; Opposite indicates the enrichment analysis results of genes with opposite expression trends in protein group and transcriptome; Protein_only indicates that there is no expression in transcriptome, and the enrichment analysis results of differentially expressed genes in protein group; Transcript_only indicates that there is no expression in protein group, and the enrichment analysis results of differentially expressed genes in transcriptome

The results of the GO enrichment analysis showed that the two groups of differential genes and proteins were mainly enriched in cellular components, molecular functions, and biological processes. The top 10 genes and proteins in the two groups are shown in Fig. 5 A and 5B. Similarly, the KEGG enrichment analysis was performed for the top 10 differentially expressed pathways with the most significant enrichment degree among the genes and proteins that are associated with LUAD (Fig. 5 C and 5D). Then, the differentially expressed genes and proteins were integrated and analyzed. There were 206 overlapping distributions between 2,814 differential genes and 470 differentially expressed proteins (Fig. 5E). The significance level of the top 10 biological process entries in the four types of association analysis results of the proteome and transcriptome based on GO and KEGG enrichment are shown in Fig. 6 A and 6B, respectively. The results showed that the gene enrichment pathways with the same expression trend of proteome and transcriptome were mainly enriched in neutral amino acid transport, response to viruses, the integrin-mediated signaling pathway, amino acid transport, the p53 signaling pathway, etc. The results indicated that Ttc39c might be involved in the progression of lung adenocarcinoma through these pathways.

### High-resolution non-target metabonomics analysis of Ttc39c and its metabolites in LUAD

**Fig. 7:**
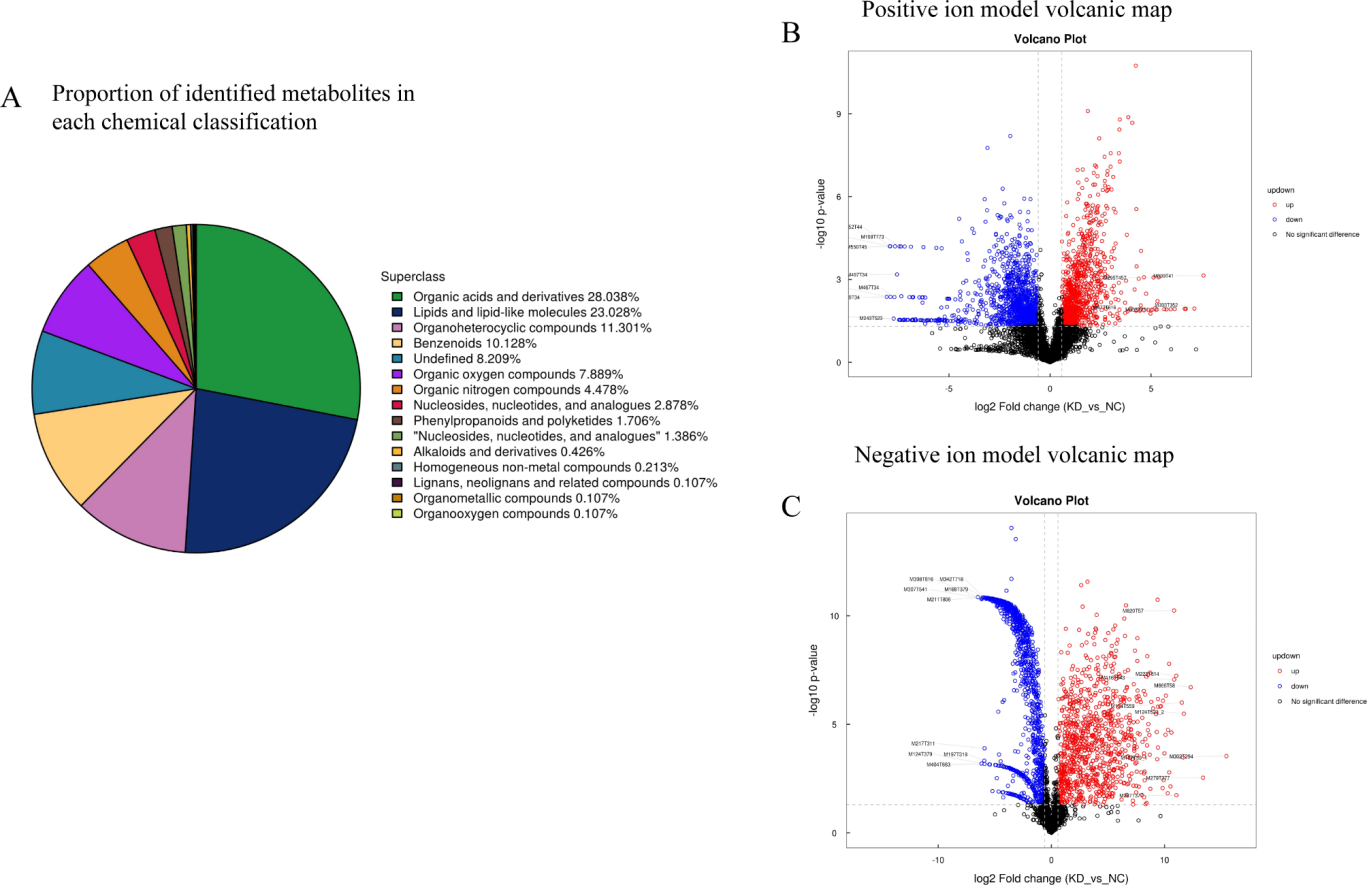
Metabonomics analysis of Ttc39c and its metabolites in LUAD. (A) Metabolic chemical classification chart. The color blocks of different colors in the figure express different chemical classification items, and the percentage represents the percentage of metabolites in the chemical classification items in all identified metabolites. Metabolites without chemical classification are defined as undefined. (B) Positive ion model volcanic map of top ten metabolites with significant differences. (C) Negative ion model volcanic map of top ten metabolites with significant differences. The abscissa is the logarithmic value of log2 of fold change; The ordinate is the logarithmic value of -log10 of significance *p* value. Significant metabolites: metabolites with FC > 1.5 and *p* value < 0.05 are expressed in rose red, metabolites with FC < 0.67 and *p* value < 0.05 are shown in blue. Non-significantly different metabolites are indicated in black. The metabolites marked in the figure are TOP 10 with up-regulated expression change (FC) and TOP 10 with down-regulated expression change in metabolites with qualitative names

**Fig. 8:**
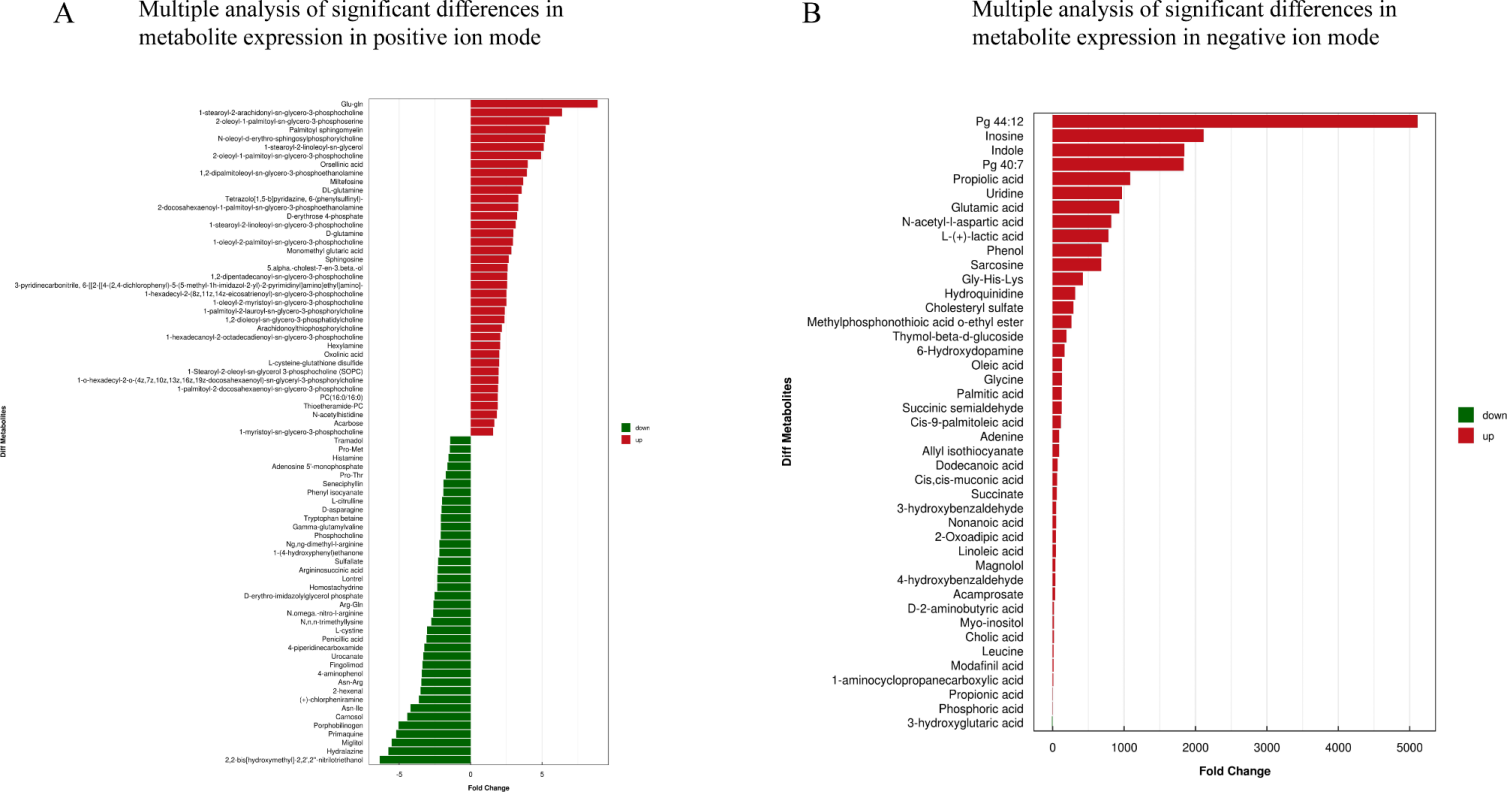
Metabonomics analysis of Ttc39c and its metabolites in LUAD. (A) Histogram of difference multiple analysis of metabolites expression with significant difference in positive ion mode. (B) Histogram of difference multiple analysis of metabolites expression with significant difference in negative ion mode. The abscissa indicates the differential expression multiple, red indicates that the differential expression multiple is greater than 1, and green indicates that the differential expression multiple is less than 1. The ordinate indicates metabolites with significant difference

**Fig. 9:**
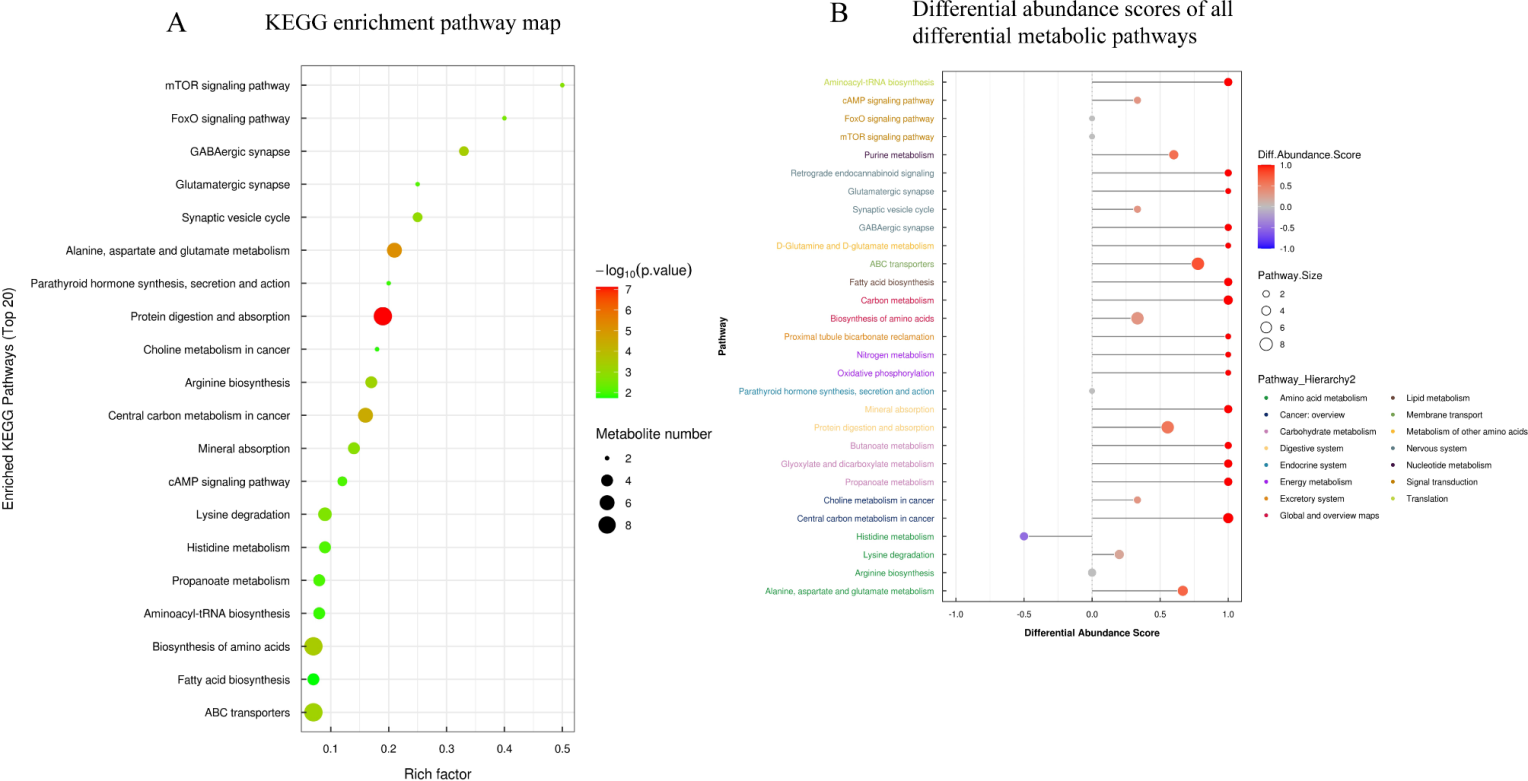
Metabonomics analysis of Ttc39c and its metabolites in LUAD. KEGG pathway enrichment (Top 20 metabolic pathways with the highest significance). The bigger the bubble, the bigger the influence factor; The color of bubbles indicates the *P* value of enrichment analysis. The darker the color, the smaller the P value, and the more significant the enrichment degree. Rich factor represents the proportion of the number of differential metabolites in this pathway to the number of metabolites annotated in this pathway. (B) Differential abundance scores of all differential metabolic pathways (classification and attribution according to Pathway_Hierarchy). Y-axis represents the name of the differential pathway, and X-axis coordinates represent the differential abundance score. DA score is the total change of all metabolites in metabolic pathway. A score of 1 indicates that the expression trend of all identified metabolites in this pathway is up-regulated, and − 1 indicates that the expression trend of all identified metabolites in this pathway is down-regulated. The length of the line segment indicates the absolute value of DA score, the dot size at the end of the line segment indicates the number of metabolites in the pathway, and the larger the dot, the more metabolites. It is proportional to the depth of dot color and DA score value. The darker the red color is, the more the expression of this channel tends to be up-regulated. The darker the blue color is, the more it tends to be down-regulated

The metabolomics method based on UHPLC-Q-TOF MS was used to analyze the changes in the metabolic profile. The positive and negative Hotelling’s T2 test results of the overall samples showed that the QC samples were within the 99% confidence interval. This indicated that the repeatability of the experiment was good (Additional Fig. 2 A and 2B). All metabolites identified by combining positive and negative ions were classified and counted based on their chemical taxonomy. The proportion of various metabolites is shown in Fig. 7 A. The results showed that the metabolites mainly accumulated in organic acids and their derivatives, lipids, and lipid-like molecules. The significant differences in the metabolites between the groups were analyzed by multidimensional statistics. The positive and negative volcano maps showed the top 10 upregulated and top 10 downregulated genes selected for labeling (Fig. 7B C). The Variable Importance in Projection (VIP) scores obtained in the OPLS-DA model were used to measure the influence intensity and interpretation ability of the expression mode of each metabolite on the classification and discrimination of each group of samples for mining biologically significant differential lipid molecules. The histogram showed a significant difference and a change of metabolic difference multiple (Fig. 8 A and 8B). Among them, PG 44; 12. The difference between Glu, Gln, and 2,2-bis (hydroxymethyl)-2,2,2-nitrotriethanol was the most significant. Further enrichment analysis of the top 20 differential metabolic pathways was conducted based on the KEGG pathway mapper function (Fig. 9 A). The metabolic changes in significantly enriched metabolic pathways were analyzed by differential abundance scores (Fig. 9B). The differential metabolites were mainly concentrated in carbohydrate metabolism, energy metabolism, central carbon metabolism in cancer, etc.

## Discussion

Identifying oncogenic driver gene mutations and their application in predicting the response to targeted therapy are the main techniques for clinicians to diagnose and treat lung adenocarcinoma [22]. The main targets of the targeted therapeutic drugs include EGF, ALK, ROS1, BRAF, RAS, etc., which have greatly benefitted individualized treatment of patients; however, the drug resistance rate is increasing [23, 24]. Immunotherapy has become popular for cancer treatment, including LUAD treatment [25–27]. The most studied checkpoint receptors in tumor immunotherapy targets are cytotoxic T lymphocyte antigen-4 (CTLA-4) and the programmed death-1 (PD-1) receptor, which downregulates the activation, proliferation, and function of T cells through different mechanisms [28, 29]. However, the effective rate of single drug immunotherapy is low, and combined immunotherapy is generally required. Therefore, a new target needs to be urgently identified to reduce the drug resistance rate of lung adenocarcinoma and improve the therapeutic effect.

The protein encoded by the Ttc39c gene contains several putative tetrapeptide repeat (TPR) domains, which are structural motifs that facilitate protein interaction [18]. Ttc39c strongly influences muscle cell signal transduction and differentiation. It participates in skeletal muscle atrophy by regulating ERK1/2 MAP kinase and hedgehog signal transduction [30]. Furthermore, zebrafish embryos with knocked down Ttc39c show various fiber disease phenotypes, including abnormal body bending and abnormal organogenesis [31]. These findings suggested that Ttc39c might be closely related to cell heterogeneity. To determine the role of Ttc39c in the progression of LUAD, we analyzed the RNA-seq data (FPKM values) from The Cancer Genome Atlas (TCGA) database. The results showed that the expression of Ttc39c increased significantly in the LUAD tissues compared to that in the normal tissues. The results also showed that patients with high Ttc39c expression had a worse overall survival (OS) than the patients with low Ttc39c expression. We also studied the function of Ttc39c in the lung cancer cells A549 and NCI-H1299. The proliferation, metastasis, and cell cloning ability of the A549 and NCI-H1299 cells decreased after the Ttc39c gene was deleted, but the apoptosis of the cells increased. These results suggested that Ttc39c strongly influences the pathological progression of LUAD.

Transcriptome sequencing is performed to study all mRNA transcribed by specific tissues or cells at a certain period based on the Illumina sequencing platform. It helps to elucidate gene function and structure and enhances the understanding of the development of organisms and the occurrence of diseases [32]. In the joint analysis of proteome and transcriptome, the correlation analysis and the GO/KEGG enrichment analysis are used to conduct studies on molecular mechanisms such as tumor occurrence, development and metastasis, target screening, and drug design [33]. Metabolome technology is used to analyze the differences in the metabolic level between the experimental group and the control group and identify the different metabolites. This can help screen biomarkers and elucidate the biological processes associated with different metabolites [34, 35]. To determine the mechanism of action of Ttc39c in LUAD, the transcriptomics, proteomics, and metabolomics multidimensional analysis was performed. The mRNA and protein expression levels and the metabolites in tumor samples of the Ttc39c-knockout mice were measured comprehensively to elucidate the entire expression profile, which can be used to analyze the genes, proteins, and pathways regulated by Ttc39c. The gene enrichment pathways with the same expression trend of proteome and transcriptome were mainly enriched in neutral amino acid transport, response to viruses, the integrin-mediated signaling pathway, amino acid transport, the p53 signaling pathway, etc. An increase in the level of integrin is involved in the progression of LUAD [36, 37]. Due to an increase in the energy demand of cancer cells, large quantities of amino acids are transported to tumor cells. Amino acid transporters can be used as therapeutic targets for breast cancer [38, 39]. The involvement of the p53 cell pathway in tumor progression has been widely studied [40, 41]. The findings suggested that Ttc39c might be involved in the progression of lung adenocarcinoma through these pathways. The metabolic pathways most likely to be associated with Ttc39c include carbohydrate metabolism, energy metabolism, and central carbon metabolism in cancer cells through KEGG enrichment and the differential abundance of various metabolic pathways. This has a common set with the enrichment of transcriptome and proteomic pathways. In this study, we determined the effects of Ttc39c on the proliferation, apoptosis, cloning, and metastasis of LUAD. Based on the above-mentioned techniques and the bioinformatics analysis, the pathways involved in Ttc39c were identified. A limitation of this study was that the pathways involved were not investigated. We aim to study the specific mechanisms based on the results of the bioinformatics analysis.

## Conclusion

To summarize, our findings provided experimental evidence that Ttc39c strongly influences the proliferation and migration of lung cancer cells. The main pathways associated with Ttc39c in lung adenocarcinoma include energy metabolism and the p53 pathway.

## Electronic supplementary material

Below is the link to the electronic supplementary material.

Supplementary Material 1

Supplementary Material 2

## Data Availability

The data used to support the findings are included in the main article. And the data are available from the corresponding author upon request.

## Abbreviations

Ttc39c: The tetratricopeptide repeat domain 39 C.
LUAD: Lung adenocarcinoma.
PD-1: programmed death receptor-1.
TPR: tetrapeptide repeat.
AAbs: antibodies against tumor antigens.
Ttc14: Tetratricopeptide repeat domain 14.
VIP: Variable Importance for the Projection.
CTLA-4: cytotoxic T lymphocyte antigen-4.
TCGA: The Cancer Genome Atlas.
OS: overall survival.
