# Supplementary material for: Ttc39c is a potential target for the treatment of lung cancer
Source: BMC Pulm Med. 2022 Oct 27;22:391. doi: 10.1186/s12890-022-02173-x (PMC9615393; doi:10.1186/s12890-022-02173-x)
Supplement: Supplementary file 1 — Supplementary Material 1 [file 12890_2022_2173_MOESM1_ESM.pdf]

Additional fig.1

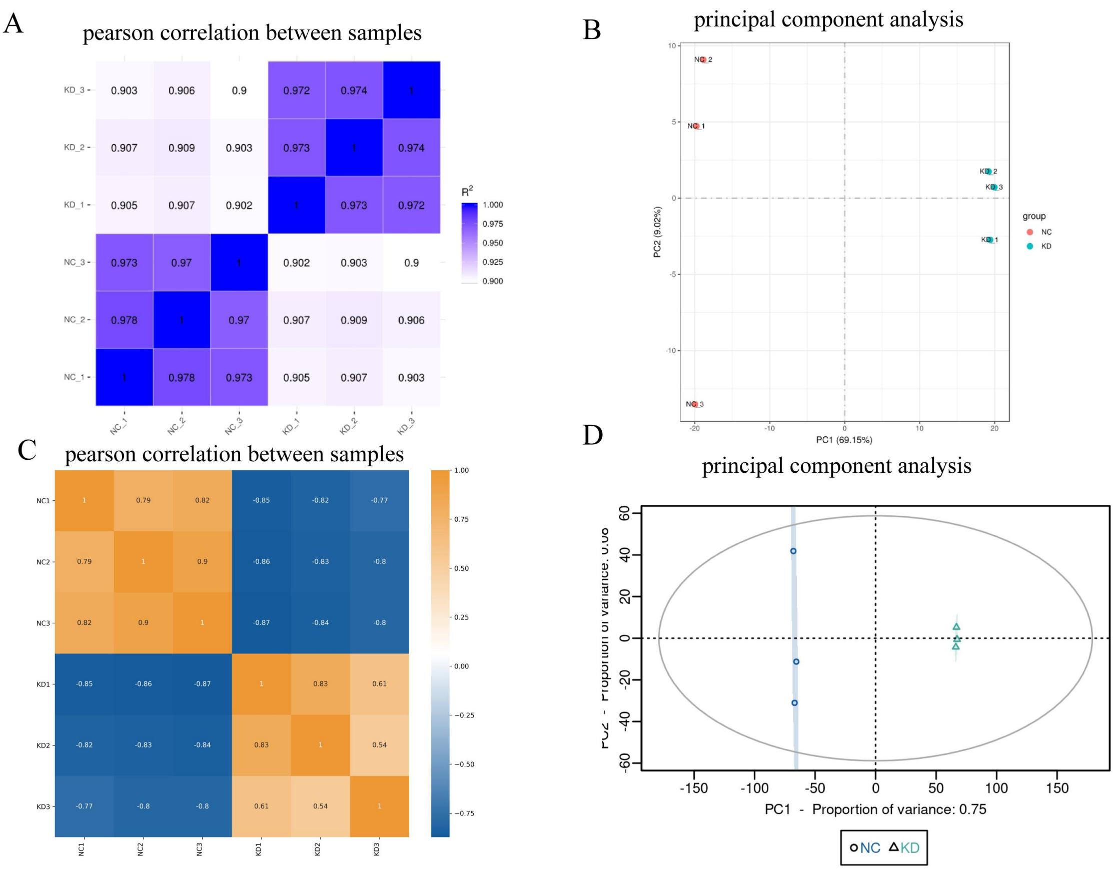

Additional fig.1. Transcriptome and proteomic analysis of lung adenoma samples from Ttc39c knockout and normal nude mice. (A) The heatmap analysis of gene expression profile. The ordinate is the square of the correlation coefficient of each sample. (B) Principal Component Analysis plots of gene between NC group and KD group. The abscissa is first principal component and the ordinate is the second principal component. (C) Pearson correlation coefficient analysis heat map of protein between NC group and KD group. Pearson coefficient is negatively correlated with -1, positively correlated with 1, and uncorrelated with 0. (D) Principal Component Analysis plots of protein between NC group and KD group. The higher the degree of aggregation between samples, the better the repeatability between samples.
