# Supplementary material for: Ttc39c is a potential target for the treatment of lung cancer
Source: BMC Pulm Med. 2022 Oct 27;22:391. doi: 10.1186/s12890-022-02173-x (PMC9615393; doi:10.1186/s12890-022-02173-x)
Supplement: Supplementary file 2 — Supplementary Material 2 [file 12890_2022_2173_MOESM2_ESM.pdf]

Additional fig.2

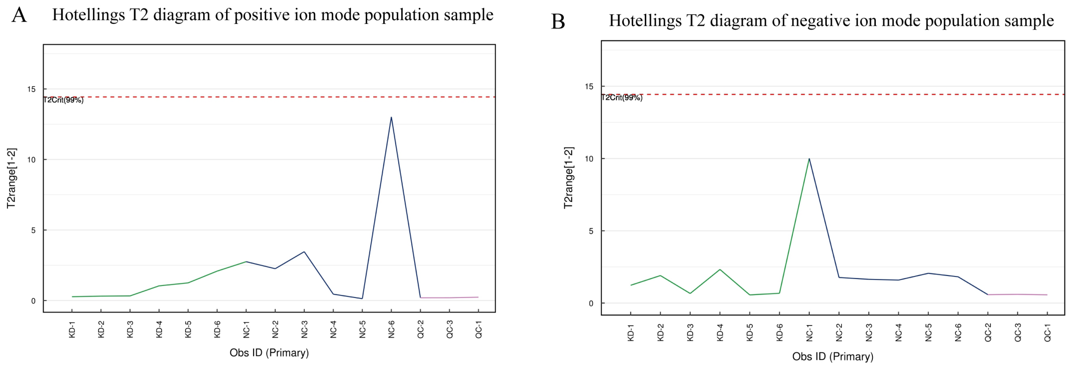

Additional fig.2. The positive and negative Hotelling's T2 test results of the overall samples. (A) Hotellings T2 diagram of positive ion mode population sample. (B) Hotellings T2 diagram of negative ion mode population sample. The abscissa represents all the experimental samples and QC samples, the ordinate reflects the confidence interval, and the red line defines the 99% confidence interval range.
